# Supplementary material for: Association between oral microbiome and seven types of cancers in East Asian population: a two-sample Mendelian randomization analysis
Source: Front Mol Biosci. 2023 Nov 21;10:1327893. doi: 10.3389/fmolb.2023.1327893 (PMC10702768; doi:10.3389/fmolb.2023.1327893)
Supplement: Supplementary file 1 [file DataSheet1.ZIP › Supplementary Materials/MR plots for tongue/tongue═╝/Lung cancer/pheno.3028_to_lung cancer_leave_one_out.pdf]

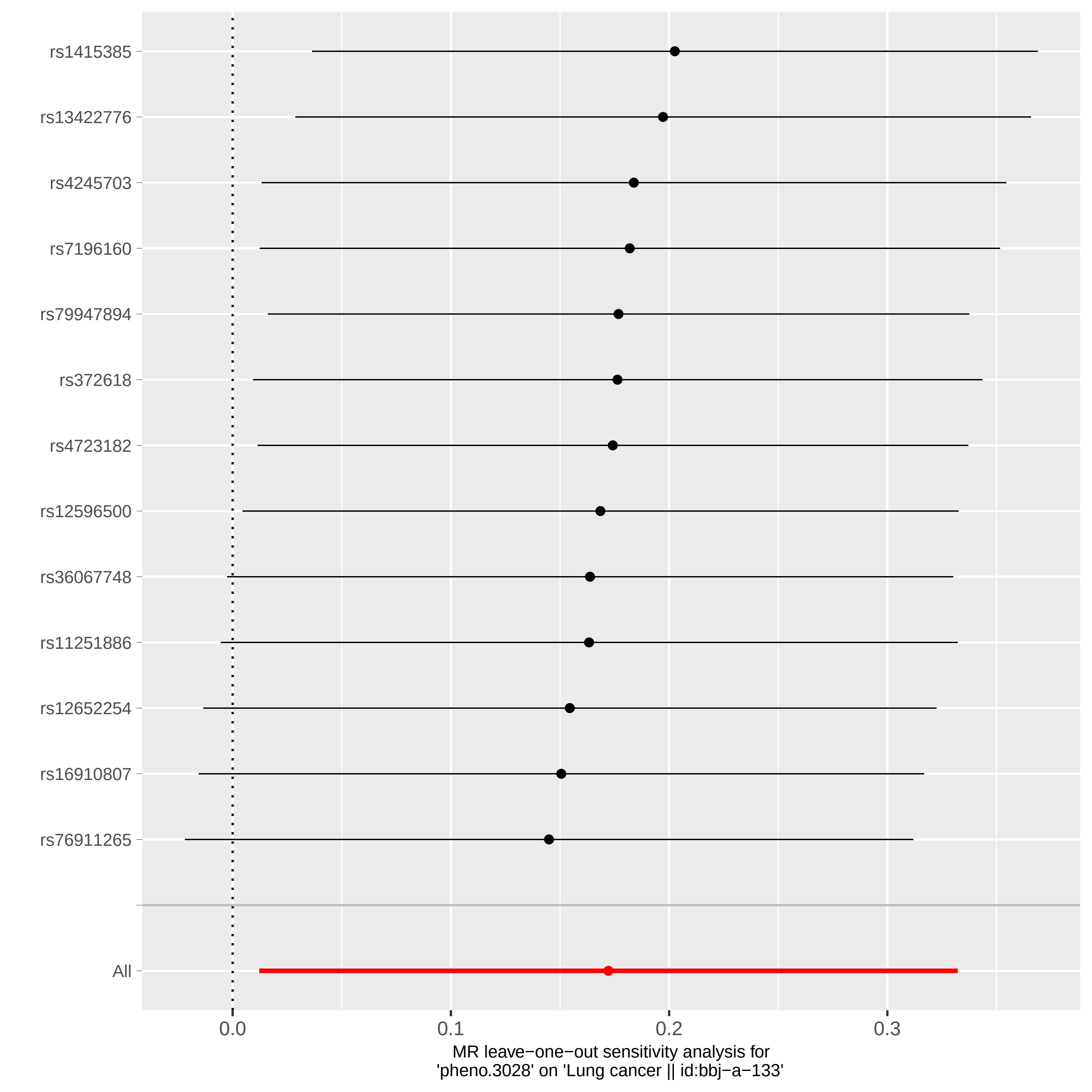

rs1415385
rs13422776
rs4245703
rs7196160
rs79947894
rs372618
rs4723182
rs12596500
rs36067748
rs11251886
rs12652254
rs16910807
rs76911265
All
0.0
0.1
0.2
0.3
MR leave−one−out sensitivity analysis for
'pheno.3028' on 'Lung cancer || id:bbj−a−133'
